# Reexamination of Statistical Methods for Comparative Anatomy: Examples of Its Application and Comparisons with Other Parametric and Nonparametric Statistics

**DOI:** 10.1155/2015/902534

**Published:** 2015-08-27

**Authors:** Roqueline A. G. M. F. Aversi-Ferreira, Hisao Nishijo, Tales Alexandre Aversi-Ferreira

**Affiliations:** ^1^Graduate School of Animal Biology, Institute of Biology, University of Brasilia, Darcy Ribeiro Campus, 70910-900 Brasília, DF, Brazil; ^2^Laboratory of Neuroscience and Behavior, Department of Physiology, University of Brasilia, Darcy Ribeiro Campus, 70910-900 Brasília, DF, Brazil; ^3^Department of System Emotional Science, Graduate School of Medicine and Pharmaceutical Sciences, University of Toyama, Toyama 930-0194, Japan

## Abstract

Various statistical methods have been published for comparative anatomy. However, few studies compared parametric and nonparametric statistical methods. Moreover, some previous studies using statistical method for comparative anatomy (SMCA) proposed the formula for comparison of groups of anatomical structures (multiple structures) among different species. The present paper described the usage of SMCA and compared the results by SMCA with those by parametric test (*t*-test) and nonparametric analyses (cladistics) of anatomical data. In conclusion, the SMCA can offer a more exact and precise way to compare single and multiple anatomical structures across different species, which requires analyses of nominal features in comparative anatomy.

## 1. Introduction

Some biological sciences are dependent on subjective decision of scientists due to lack of appropriate numerical methods to determine observations. The description of biological structures, for instance, is sometimes exhaustive and depends on scientists' subjective observations. It is well known that the ancient researchers mistook analyses of the anatomical structures including their numbers, for example, the number of the cranial nerves, probably because of the scarce conditions during the works at that time [1]. Nevertheless, most structures in the body analyzed in the past were reported to contain the same number of structures nowadays.

In fact, gross anatomy requires descriptions of qualitative variables including innervation, vascularization, origin and insertion of muscles, and arteries and nerve branches. It also requires quantitative analyses of mass, area, volume, size, and dimensional measures of such biological structures, which can be characterized by parametric statistical methods. However, parametric statistical methods can be hardly applied to description of qualitative variables in a scope of gross anatomy [2]. This is one of the reasons why the different authors with great experiences differently described structures of the same species without considering anatomical variations (for an illustrative example of confusion regarding the name and description of the tibial artery, see [3]).

Here, we propose the main methodology to characterize qualitative data in gross anatomy, which enables us to describe and compare objectively anatomical structures across different species. Specifically, there have been no adequate quantitative methods to compare discrete-nominal variables of anatomical structures. It is desirable to quantitatively assess discrete variables [4]. Indeed, a nonparametric statistical method has been proposed for comparative anatomy [2] and actually used in some works [5, 6]. Although these studies compared their nonparametric statistical method with one of nonparametric methods (chi-square), the authors did not compare their nonparametric method with parametric ones, nor did they specify possible problems of interpretation of the data. Moreover, for statistical method for comparative anatomy (SMCA), the formula must be modified so that it accepts data of multiple structures across different species.

For objective descriptions of the anatomical structures, some authors used the chi-square comparison to analyze nominal variables [7, 8] by converting frequencies of anatomical characteristics to percentages. Thus, they calculated standard deviations of the data in percentages. However, standard deviations of nonparametric data derived from a kind of the discrete categorical variables usually generate a statistical error type I [2]. Furthermore, the basis of the chi-square statistic is causality among the data, a hypothesis that is not consistent with a theory of evolution assuming the concept of the common ancestral [9]. Anatomy of body structures such as innervation, origin, and/or insertion of muscles of the arm, for instance, seems to be not random in animals that evolved from a common ancestral animal, since the ancestral animal provided basic structures and could generate derivative features in descendant animals (for a detailed review, see [2]). Nevertheless, the chi-square statistic is an important tool among multivariate analyses of discrete variables that are considered to be independent in quantitative psychology [10]. Another nonparametric method (cladistics) was applied to comparative anatomy to analyze primitive and derivative features in evolution [11, 12] but has not yet been applied to descriptive anatomy.

In this paper we compared the nonparametrical method for SMCA [5, 6] with another parametric method (*t*-test) analyzing means of the samples, using the previously published data [6]. The SMCA including Comparative Anatomy Index for groups of structures (GCAI) that enables comparison of multiple structures [5] (see Section 3.2 for details) was also compared with another nonparametric (cladistics) method.

## 2. Material

This work reanalyzed the previously published data in comparative anatomy statistic (SMCA) [5] to verify this method in detail [2] and applied the new formula (GCAI) to compare groups of structures among different species. Furthermore, using the published data [5, 6] as examples, we show the steps to calculate the SMCA and compare between the SMCA and a parametric test (*t*-test) and also between the SMCA and other nonparametric (cladistics) methods [6].

## 3. Methods

### 3.1. Methods to Compare Samples from Same Species

The first step of this statistical method for comparative anatomy (SMCA) is to analyze the frequency based on the anatomical concept of* normality* and* variation*. “A normal structure” means that it is observed in greater than 50% of cases within the same species; therefore, the variation can be observed in less than 50% of cases [13]:

(1) $$\begin{array}{c} N=N_{ijk}=\overset{q}{\underset{i=1}{\sum }}\left(r_{v(ijk)}+n_{v(ijk)}\right), \end{array}$$

where *N* is the total number of analyzed structures of the samples, *n*_*v*_ is the number of structures with variation, and *r*_*v*_ is the number of normal structures (*N* − *n*_*v*_). The subscript (*i*) indicates specific species such as humans,* Cebus*, and baboon, while the subscript (*j*) indicates specific structures (flexor pollicis longus, pronator quadratus, etc.), and the subscript (*k*) indicates parameters of the specific structures. For muscle studies, the parameters should include at least the following 4 parameters: (1) innervation, (2) origin, (3) insertion, and (4) vascularization of muscles. For example, in case of the flexor pollicis longus muscle (*j* = 1) in* Cebus *(*i* = 1), the data analyses in this step should be performed in terms of the following 4 parameters: the (1) innervation (*r*_*v*(111)_, *n*_*v*(111)_), (2) origin (*r*_*v*(112)_, *n*_*v*(112)_), (3) insertion (*r*_*v*(113)_, *n*_*v*(113)_), and (4) vascularization (*r*_*v*(114)_, *n*_*v*(114)_). Furthermore, (5) number of muscles (*r*_*v*(115)_, *n*_*v*(115)_) and (6) shape (*r*_*v*(116)_, *n*_*v*(116)_) could be added for more detailed analyses. In addition, further detailed parameters (subscript (*h*)) could be added.

The relative frequency (RF = *P*_*ijk*_) of normal structures in each parameter against the total number of structures is defined as follows:

(2) $$\begin{array}{c} \text{RF}=P_{ijk}=\frac{r_{v(ijk)}}{N}. \end{array}$$

When structures are pair, *N* will be the number of individuals multiplied by 2. It is also possible to calculate *P*_*ijk*_ in separate pieces of bodies, as well. Although any values can be used as *N*, smaller number of *N* will result in lower statistical power. Normal structure in each parameter means 0.5 < *P*_*ijk*_ ≤ 1 in practical terms. However, in mathematical ones with normality concept, *P*_*ijk*_ can vary as follows: 0 ≤ *P*_*ijk*_ ≤ 1.

In the same species, *P*_*ijk*_ is usually greater than 0.5. However, in comparison among different species, normality is different among the species; for instance, in the comparison of the dorsoepitrochlearis muscle among primates and modern humans, this muscle is rarely observed in humans and approximate *P*_*ijk*_ is 0.05 [14], while, in nonhuman primates, the dorsoepitrochlearis muscle is a normal feature, and the *P*_*ijk*_ is 1.00.

On the other hand, the palmaris longus could be defect in humans [15] and its prevalence is around 90% [16]; therefore, *N* might be 90% of total individuals. Thus, to calculate innervation, vascularization, origin, or insertion of the palmaris longus, only 90% receives attention and data from the remaining 10% are discarded. Such case is common in comparative studies, where, usually, only data in specific species are studied. Furthermore, some muscles have more than one origin or insertion, as in the triceps brachii with 3 heads, and ultimately this muscle has 4 heads of origin in modern humans [13, 15, 16]. In this case, just 2 types of the origin are observed: type 1 with 3 heads that is the normal feature and type 2 with 4 heads that is a variation.

For a more detailed analysis, it is required to calculate *P*_*ijk*_ by including such parameters in muscle, nerves, bones, arteries, and so forth. For instance, in muscle studies, the parameters have to be chosen according to the goal of researches; parameters for muscle studies should include, at least, (1) innervation (*P*_*ij*1_), (2) origin (*P*_*ij*2_), (3) insertion (*P*_*ij*3_), and (4) vascularization (*P*_*ij*4_). Furthermore, (5) number of muscles (*P*_*ij*5_) and (6) shape (*P*_*ij*6_) could be added for more detailed analyses. It is noted that small number of parameters means that the studied structure is less characterized. For instance, in case of contrahentes muscles, the number of the muscles must be analyzed because they show variation within the same species and in different species of primates as well [14].

The calculation of *P*_*ijk*_ is the first step in the SMCA analysis, and single *P*_*ijk*_ could be compared among different species. However, what we are seeking is comparison of multiple *P*_*ijk*_ among different species. The next step is to specify pondered values for coefficients (i.e., weighted coefficients) (*w*_*k*_) that are multiplied by *P*_*ijk*_. The coefficients must be determined based on anatomical perspective; a parameter for a specific feature with small *P*_*ijk*_ is not important when we assess similarity of a given structure. That is, since the small *P*_*ijk*_ is ascribed to greater number of variations, small *P*_*ijk*_ must accompany small weighted coefficient, while large *P*_*ijk*_ (i.e., small number of variations) must accompany greater weighted coefficients.

We gave the weighted coefficient 3 to* innervation *(*k* = 1, *w*_1_ = 3) in case of muscles. When the muscles are formed during the development of animals, a specific nerve terminates on a specific muscle [17]. Thus, variation in nerve innervation of muscles is small, and a variation of innervation is highly sensitive to differences among different individuals within the same species as well as among different species. Among the 4 parameters noted above (innervation (*k* = 1), origin (*k* = 2), insertion (*k* = 3), and vascularization (*k* = 4)), origin and insertion usually show similar variations. Thus, the both weighted coefficients should receive the same weight coefficient 2 (*w*_2_ = 2 for origin and *w*_3_ = 2 for insertion). Finally, the parameter with greater variation, vascularization (*k* = 4), received the weighted coefficient 1 (*w*_4_ = 1). Indeed, vascularization can be different between the same muscles in bilateral sides within the same individuals [18].

Zero cannot be accepted as weighted coefficient (*w*_*k*_). Therefore, *w*_*k*_ must be greater than zero; that is, *w*_*k*_ > 0. To make the calculation easier and to keep clear parameters, the best choice is to use only integer values; that is, *w*_*k*_ ≥ 1. Based on the above inference, an important rule here is clear; when the weighted coefficients are defined, the values should depend on different degree of variation (highest value to the weighted coefficient for the parameter with the lowest degree of variation or the same value to weighted coefficients for the parameters with same degree of variation). The values also should be discrete since it is difficult to find proportional values that represent exact difference among nominal variables. Thus, the best way is to choose integer values according to variations of studied features.

After designation of pondered values for weighted coefficients and calculation of *P*_*ijk*_, the next step for SMCA is to calculate the Pondered Average of Frequencies (PAF = *P*_*w*(*ij*)_), according to the following formula:

(3) PAF=Pw(ij)=∑k=1qwk·Pijk∑k=1qwk;for  any  species  i=1,2,…,s,any  muscles  j=1,2,…,m,

where *P*_*ijk*_ is the relative frequency and *w*_*k*_ is the weighted coefficient attached to a given parameter. For example, in muscle 1 of species 1, *P*_111_ is relative frequency of innervation, and weighted coefficient *w*_1_ is 3; *P*_112_ is relative frequency of the muscle origin, and *w*_2_ is 2; *P*_113_ is relative frequency of muscle insertion, and *w*_3_ is 2; and *P*_114_ is relative frequency of vascularization, and *w*_4_ is 1 [5].

In practical terms, *P*_*w*(*ij*)_ must be greater than 0.5 and less than or equal to 1; that is, 0.5 < *P*_*w*(*ij*)_ ≤ 1. In fact, *P*_*w*(*ij*)_ could be 1 if every *P*_*w*(*ij*)_ has maximal value 1, and if every *P*_*w*(*ij*)_ is minimum (*P*_*w*(*ij*)_ > 0.5), the *P*_*w*(*ij*)_ will be 0.5, as well. In mathematical terms, again, regardless of concept of normality, *P*_*w*(*ij*)_ can vary within the range of 0 ≤ *P*_*w*(*ij*)_ ≤ 1, since *P*_*w*(*ij*)_ could be zero or less than 0.5 in case of the analyses among different species. The value of *P*_*w*(*ij*)_ can be used to assess quantitative difference among studied structures in that equal values indicate high similarity and large difference in the values between two species indicates dissimilarities or less similarity.

### 3.2. Methods to Compare Different Structures of the Same Species and among Different Species

To compare structures among different species or different structures in the same species, *P*_*w*(*ij*)_ has to be calculated in each structure in each species and the *P*_*ijk*_ must be estimated in comparison with the data of reference species (*control species*). For instance, the corachobrachialis muscle has one or two heads of origin depending on species of primates [17]. In case of this muscle, *P*_*ijk*_ (relative number of heads) could be different according to the number of heads in the* control species*. Thus, before calculating *P*_*w*(*ij*)_, it is important to make sure that the *P*_*ijk*_ must be consistently calculated in comparison with* control species* (see below).

For example, the maximum number of types of origin is 2 in the corachobrachialis (*j* = 1); type 1 has one origin and type 2 has two origins (*k* = 2). *P*_*ijk*_ could take different values according to the number of heads in the reference species (*control species*) (*i* = 1). For example, for noncontrol species to be studied (*i* = 2), the *P*_212_ of type 1 (number of origin is 1) will be 1 in reference to the species with 1 head, and *P*_212_ of type 1 will be 0.5 in reference to the species with 2 heads. In case of the muscle that has 1 to 3 heads of origins across different species, the *P*_*ijk*_ value should be divided by maximum number (i.e., 3) of heads (1/3 = 0.333), since *P*_*ijk*_ should not be greater than 1. Thus, when* control species* with 3 heads of origin is reference, *P*_212_ in species with 3 heads (*i* = 2) is 1.000, *P*_312_ in species with 2 heads (*i* = 3) is 0.667 (2/3), and *P*_412_ in species with 1 head (*i* = 4) will be 0.333 (1/3).

It is also important that the values of *P*_*ijk*_ should be obtained firstly in the* control species*. If the* control species *(*i* = 1) have normally two heads of origin (*k* = 2) in the corachobrachialis (*j* = 1) and if 100% of individuals in this species have two heads of origin, *P*_112_ in this species will be 1. In the case wherein 90% of individuals in this species have two heads, *P*_112_ will be 0.9. In other noncontrol species (*i* = 2) in which the normal is one head of origin of the corachobrachialis, if 100% of individuals of the studied sample have one head, the *P*_212_ will be 0.5, and if 90% of the samples have one head, *P*_212_ will be 0.45. These values are used to estimate *P*_*w*(*ij*)_, which will be applied to the CAI analyses (see below).

Although any species can be used as* control species*, the species studied in the first time or the species with much known data should be chosen as* control species*. To compare any single structure (e.g., muscle) between two different species (*i* ≠ *i*′), the data in any noncontrol species can be compared one by one with those in the* control species* using the Comparative Anatomy Index (CAI) defined by the following formula:

(4) $$\begin{array}{c} \text{CA}{\text{I}}_{ii^{\prime }}=\left|P_{w(ij)}-\right.\left.P_{w(i^{\prime }j)}\right|,\;\text{where}\;i\neq i^{\prime }.\; \end{array}$$

The CAI_*ii*′_ represents an absolute difference of weighted averages (*P*_*w*(*ij*)_) of a single structure between the* control* (*i*) and other noncontrol (*i*′) species. To compare one structure (*j* = 1) with one parameter (*k* = 1) between the control (*i* = 1) and noncontrol (*i*′ = 2) species, the formula can be modified as follows:

(5) $$\begin{array}{c} \text{CA}{\text{I}}_{12}=\left|P_{w(11)}-P_{w(21)}\right|,\;\text{where}\;i=1,\;\;i^{\prime }=2. \end{array}$$

It is noted that the CAI_*ii*′_ ranges from 0 to 1; that is, 0 ≤ CAI_*ii*′_ ≤ 1. This is because the maximum value of *P*_*w*(*ij*)_ is 1 and the minimum is 0. Note that this equation permits only comparison of just one structure between the 2 species. However, the SMCA analysis of the muscles in the forearm [5] reported necessity to compare multiple muscles among different species, for example, to compare groups of the deep flexor muscles in the forearm among different species, because these muscles work together for a common function. Comparisons of them as a group would indicate similarities in relation to functions, phylogeny, and taxonomy. Thus, the authors [5] suggested the GCAI to compare a group of the muscles among species, one by one based on the sum of the *P*_*w*(*ij*)_, as follows:

(6) Pw(i)=∑j=1mjPw(ij)mj;for  any  species  i=1,2,…,s,studied  structures  j=1,2,…,m,

and *m*_*j*_ is the number of studied structures (e.g., muscles); indeed, *m*_*j*_ = *m* because the same number of structures is mostly studied in each species.

The GCAI, which represents difference in *P*_*w*(*i*)_ based on multiple muscle structures between the* control* (*i*) and other noncontrol (*i*′) species, is defined by the following formula:

(7) $$\begin{array}{c} \text{GCA}{\text{I}}_{ii^{\prime }}=\left|P_{w(i)}-P_{w(i^{\prime })}\right|, \end{array}$$

or

(8) $$\begin{array}{c} \text{GCA}{\text{I}}_{ii^{\prime }}=\left|\frac{\sum_{j=1}^{mj}P_{w(ij)}}{m_{j}}-\frac{\sum_{j=1}^{mj}P_{w(i^{\prime }j)}}{m_{j}}\right|. \end{array}$$

Based on the above inferences, using SMCA, the values close to 0.000 suggest high similarity of the structures between the species, and the value 1.000 indicates that those are completely different structures. Thus, the GCAI is the absolute difference in mean weighted averages of *P*_*w*(*ij*)_ for multiple muscles between the two species and is defined in Table 1.

## 4. Results

### 4.1. Examples of Application and Calculation of the SMCA

We reanalyzed the previous data of the anatomical structures in comparative anatomy by application of the SMCA. The statistic for comparative anatomy was applied to the muscle extensor pollicis brevis in the forearm to compare the muscles among primates (*Cebus* (Ce) (now* Sapajus*), baboons (Ba), chimpanzees (Ch), and modern humans (Hu)), and Ce was used as the* control species* [5]. According to the data by Aversi-Ferreira et al. [5], the characteristics of this muscle are shown in Table 2.

Firstly, the* control species* was* Cebus* (Ce), and the *P*_*ijk*_ was calculated for the muscle extensor pollicis brevis using parameters of innervation, origin, insertion, and vascularization. Eight samples of Ce were used; therefore, 16 muscles were analyzed. All specimens contained the muscle extensor pollicis brevis, and all studied muscles showed same innervation (*P*_111_) and vascularization (*P*_114_), identical origin (*P*_112_), and insertion (*P*_113_).

For the innervation of the muscle in Ce, relative frequency *P*_111_ is as follows:

(9) $$\begin{array}{c} \text{RF}=P_{111}=\frac{r_{v(111)}}{N}=\frac{16}{16}=1. \end{array}$$

The same analysis must be done for each parameter in each muscle of different species. It is noted that the parameters in primates other than Ce were obtained from previous literatures [15, 17, 18]. The insertion in Hu (*i* = 2) does not match with that in Ce (Table 2). Then, the *P*_213_ for the parameter insertion (*k* = 3) is zero. In Ba (*i* = 4), every *P*_41*k*_ will be zero, because this muscle is absent in this species.

The next step is to calculate the *P*_*w*(*ij*)_ in each species. The *P*_*w*(11)_ of Ce is as follows:

(10) Pw(11)=∑k=1qwk·Pijk∑k=1qwk=1·3+1·2+1·2+1·18=1.000.

In Hu, the *P*_(213)_ is zero, and then the *P*_*w*(21)_ is as follows:

(11) Pw(21)=∑k=1qwk·Pijk∑k=1qwk=1·3+1·2+0·2+1·18=68=0.750.

The *P*_*w*(31)_ for Ch (*i* = 3) is equal to *P*_*w*(11)_. In Ba (*i* = 4), since the extensor pollicis brevis is absent, *P*_*w*(41)_ is zero. The conclusion is *P*_*w*(11)_ = *P*_*w*(31)_ = 1.000, *P*_*w*(21)_ = 0.750, and *P*_*w*(41)_ = 0.000.

The next step is to calculate the CAI between the* control species* and others, one by one, as follows:

(12) $$\begin{array}{c} \text{CA}{\text{I}}_{12}=\left|P_{w(11)}-P_{w(21)}\right|=\left|1.000-0.750\right|=0.250;\\ \text{CA}{\text{I}}_{13}=\left|P_{w(11)}-P_{w(31)}\right|=\left|1.000-1.000\right|=0.000;\\ \text{CA}{\text{I}}_{14}=\left|P_{w(11)}-P_{w(41)}\right|=\left|1.000-0.000\right|=1.000. \end{array}$$

The results by the CAI calculation represent degree of difference in the extensor pollicis brevis among the species. The CAI values suggest that Ce and Ch have identical extensor pollicis brevis (high similarity), while Ce and Hu show some differences (somewhat similar), and Ce and Ba are highly different. It is because the extensor pollicis brevis does not exist in Ba or that muscle have no match between Ce and Hu. These results provide quantitative measures with previous papers that described the differences in the extensor pollicis brevis among different species.

It is also important to verify usefulness of the GCAI. We reanalyzed the previous data [5] and applied CAI to individual deep flexor muscles in the forearm (Table 3) and GCAI to the group of the deep flexor muscles in the forearm (Table 4). Although CAIs indicate the difference in individual muscles, it is unknown how the deep flexor muscles in the forearm as a group are different between the species. The GCAI values suggest that the flexor deep muscles in the forearm of the* Cebus* (Ce) are more similar to, in order, chimpanzee, modern humans, and baboon (Table 4).

### 4.2. Comparison of the SMCA with Parametrical Method (*t*-Test)

A previous study analyzed the palmaris longus among 9 nonhuman primates and humans [6] by calculating ratios of muscle length to its tendon length, and these data were submitted to *t*-test. Additionally, the characteristics of these muscles were compared among the 9 species by the SMCA (Table 5) (data from* Aotus* and modern humans were not used; for detailed analysis, see [6]). Using CAI (SMCA), nominal variables of features in the palmaris longus including (1) origin, (2) insertion, (3) innervation, (4) muscle presence, and (5) type of belly arrangement were analyzed.

The results by parametric analysis of the ratios (muscle length/tendon length) are consistent with the primate grouping (distinction of apes, old world primate, new world primates, and prosimii); within the same group, the ratios were not significantly different (Table 5). These data are further consistent with those by CAI (SMCA) analysis of the palmaris longus (Table 5). These parametric data are at least partially consistent with those by the nonparametric (CAI) comparison (Table 6).

### 4.3. Comparison of the SMCA with Other Nonparametric Methods

Another possibility to study nominal variables is to use the cladistics method. This method considers the binary characters and any other possibility could be an error, because these features are mutually excluded [11]. Indeed this characteristic limits its application to morphological analyses of structures since it considers just two parameters: 0 for absent characteristic and 1 for its presence. Nevertheless, this method is important in evolutionary studies, since this method might provide a different concept; the cladistics analysis prioritizes the primitive and derivative features [12], while the morphological analysis prioritizes utmost characters observed in a structure.

To compare cladistics and SMCA methods, cladistics and CAI were applied to the same data in Table 2; the results are shown in Table 7.

CAI in Table 7 indicated that the pronator quadratus is similar among Ce, Ch, and Ba and different between Ce and Hu. The cladistics analysis also indicated the same results. For the flexor digitorum profundus, CAI indicated that this muscle is similar between Hu and Ce, somewhat similar between Ch and Ce, and completely different between Ba and Ce. The cladistics analysis also indicated the same characteristics; Hu, Ch, and Ce shared the same features of this muscle but were totally different from Ba. For the flexor pollicis longus, CAI indicated this muscle is identical in Ce and Ch, somewhat similar between Hu and Ce, and highly different between Ba and Ce. The cladistics analysis indicated that this muscle was similar between Ce and Ch and different between Hu and Ce and between Ba and Ce. Thus, the results by both methods are consistent. Nevertheless, CAI provides quantitative data to assess the features. Furthermore, GCAI provides further information demonstrating that the group of these muscles of Ce was more similar to Ch, Hu, and Ba in this order (Table 7).

## 5. Discussion

Firstly, an accurate analysis is fundamental in comparative anatomy. Photographs of structures can be used to verify the new data mainly when the new data disagree with others. However, numerical methods can help to avoid subjectivities and mistakes and make it easier to assess group similarities and to observe differences among structures and specimens. The application of numerical methods to comparison of biological structures could avoid prolix texts and ensure more exact and precise conclusions on similarities or differences among biological structures. This is especially true for gross anatomy where the nominal variables are studied and described.

Nonparametric statistics are less exact than parametric ones [19]. Numerical parametric analyses can be performed with observation of frequency of characteristics based on fundamental concepts of normality and variation in anatomy. However, the nonparametric methods take advantage when parametric methods cannot be used in situations where nominal variables are included in the analysis and the measured data do not match nonparametric one. That is, parametric statistics are preferable when continuous variables are available [19], while nonparametric methods could be applied to nominal variables. As shown in Table 5, both methods match partially when the same samples were analyzed by both methods [20].

The analyses by nonparametric methods (chi-square and cladistics) indicate that cladistics might be more applicable to anatomical analysis. However, this method cannot analyze frequency of features. Furthermore, chi-square method also has a problem in its basic premises; causality of data used in this method disagrees with evolution theory assuming a common ancestral [9], and it is not easy to obtain numerical nominal data from expected and observed data. Although the results by cladistics and CAI are consistent (Table 7), it is noted that CAI provides quantitative data to assess features. Furthermore, GCAI provides additional information of a group of multiple structures.

The objective of cladistics method is different from that of SMCA; cladistics method offers solution when data from large groups are analyzed in evolution analysis, while the goal of SMCA is to obtain utmost details from anatomical structures when multiple features are analyzed. The cladistics is used in evolution analysis, especially, to obtain genealogic tree. However, it can be applied to comparative anatomy, because observation of presence and absence of a specific feature provides a general idea of similarity or difference among the structures.

In conclusion, the SMCA can offer more exact and precise method to compare structures to assess same or different groups, which requires analyses of nominal features in comparative anatomy.

## Figures and Tables

**Table 1:** Illustration of individual steps to compute GCAI based on multiple muscles.

Species (*i* = 1,2,…, *s*)		Species 1 (*control species*) (*i* = 1)				Species 2 (*i* = 2)				⋯	Species *s* (*i* = *s*)			

Investigated structures (*j* = 1,2,…, *m*)		*M*_1_	*M*_2_	⋯	*M*_*m*_	*M*_1_	*M*_2_	⋯	*M*_*m*_	⋯	*M*_1_	*M*_2_	⋯	*M*_*m*_
*P*_*ijk*_	Specific weights given to variation	_ _	_ _	_ _	_ _	_ _	_ _	_ _	_ _	_ _	_ _	_ _	_ _	_ _
Innervation (*k* = 1)	*w*_1_ (=3)	*P*_111_	*P*_121_	⋯	*P*_1*m*1_	*P*_211_	*P*_221_	⋯	*P*_2*m*1_	⋯	*P*_*s*11_	*P*_*s*21_	⋯	*P*_*sm*1_
Origin (*k* = 2)	*w*_2_ (=2)	*P*_112_	*P*_122_	⋯	*P*_1*m*2_	*P*_212_	*P*_222_	⋯	*P*_2*m*2_	⋯	*P*_*s*12_	*P*_*s*22_	⋯	*P*_*sm*2_
Insertion (*k* = 3)	*w*_3_ (=2)	*P*_113_	*P*_123_	⋯	*P*_1*m*3_	*P*_213_	*P*_223_	⋯	*P*_2*m*3_	⋯	*P*_*s*13_	*P*_*s*23_	⋯	*P*_*sm*3_
Vascularization (*k* = 4)	*w*_4_ (=1)	*P*_114_	*P*_124_	⋯	*P*_1*m*4_	*P*_214_	*P*_224_	⋯	*P*_2*m*4_	⋯	*P*_*s*14_	*P*_*s*24_	⋯	*P*_*sm*4_
Weighted averages for single muscle (PAF = *P*_*w*(*ij*)_)		*P*_*w*(11)_	*P*_*w*(12)_	⋯	*P*_*w*(1*m*)_	*P*_*w*(21)_	*P*_*w*(22)_	⋯	*P*_*w*(2*m*)_	⋯	*P*_*w*(*s*1)_	*P*_*w*(*s*2)_	⋯	*P*_*w*(*sm*)_
Weighted averages for multiple muscles (mean of *P*_*w*(*ij*)_) (*P*_*w*(*i*)_)		*P*_*w*(1)_				*P*_*w*(2)_				⋯	*P*_*w*(*s*)_			
GCAI = |*P*_*w*(*i*)_ − *P*_*w*(*i*′)_|						|*P*_*w*(1)_ − *P*_*w*(2)_|				⋯	|*P*_*w*(1)_ − *P*_*w*(*s*)_|			

*Note*. *P*_*ijk*_: proportion of normal structured organs, where *i* represents individual species, *j* represents individual structures (i.e., muscles), and *k* represent individual parameters of the muscles.

**Table 2:** General features of the extensor pollicis brevis muscle based on Aversi-Ferreira et al. [5]. In primates other than Ce, only the differences in muscle parameters from Ce are indicated.

Muscle	Parameters	Ce (*i* = 1)	Hu (*i* = 2)	Ch (*i* = 3)	Ba (*i* = 4)
Extensor pollicis brevis	Origin (*k* = 2)	Proximal third of the radius and interosseous membrane	Single insertion in distal phalange of the thumb	Highly similar to Ce	Absent
	Insertion (*k* = 3)	Articular capsule of the trapezoid-metacarpal I articulation and the base of this last bone			
	Innervation (*k* = 1)	Posterior interosseous nerve			
	Vascularization (*k* = 4)	Posterior interosseous artery			

**Table 3:** CAIs for the individual flexor deep muscles in the forearm to indicate degree of difference from the control species (Ce).

	Pronator quadratus	Flexor digitorum profundus	Flexor pollicis longus
*Cebus* (Ce)	Reference	Reference	Reference
Modern human (Hu)	CAI = 0.375	CAI = 0.000	CAI = 0.125
Chimpanzee (Ch)	CAI = 0.000	CAI = 0.063	CAI = 0.000
Baboon (Ba)	CAI = 0.000	CAI = 1.000	CAI = 0.250

**Table 4:** *P*_*ijk*_, *P*_*w*(*ij*)_ and GCAIs for the flexor deep muscles in the forearm to indicate degree of differences in this muscular group among different species in relation to the control species (Ce).

Species (*i* = 1,2,…, *s*)		*Cebus* (Ce), control species or reference (*i* = 1)			Modern humans (Hu) (*i* = 2)			Chimpanzee (Ch) (*i* = 3)			Baboon (Ba) (*i* = 4)		

Investigated structures (flexor deep muscles of forearm)		Pronator quadratus	Flexor digitorum profundus	Flexor pollicis longus	Pronator quadratus	Flexor digitorum profundus	Flexor pollicis longus	Pronator quadratus	Flexor digitorum profundus	Flexor pollicis longus	Pronator quadratus	Flexor digitorum profundus	Flexor pollicis longus
*P*_*ijk*_	Specific weights given with respect to variation												
Innervation (*k* = 1)	*w*_1_ (=3)	1	1	1	0	1	1	1	1	1	1	0	1
Origin (*k* = 2)	*w*_2_ (=2)	1	1	1	1	1	0.5	1	1	1	1	0	1
Insertion (*k* = 3)	*w*_3_ (=2)	1	1	1	1	1	1	1	0.5	1	1	0	0
Vascularization (*k* = 4)	*w*_4_ (=1)	1	1	1	1	1	1	1	1	1	1	0	1
Weighted averages for single muscle (PAF = *P*_*w*(*ij*)_)		1	1	1	0.625	0	0.875	0	0.9375	0	0	1	0.750
Weighted averages for multiple muscles (mean of *P*_*w*(*ij*)_) (*P*_*w*(*i*)_)		1			$\frac{0.625+1+0.875}{3}=0.833$			$\frac{1+0.9375+1}{3}=0.980$			$\frac{0+1+0.750}{3}=0.583$		
GCAI = |*P*_*w*(*i*)_ − *P*_*w*(*i*′)_|		Reference			1+1+13-0.625+1+0.8753=(1+1+1)-(0.625+1+0.875)3=0.167			$\left|\frac{\left(1+1+1)-(1+0.9375+1\right)}{3}\right|=0.020$			$\left|\frac{\left(1+1+1)-(0+1+0.750\right)}{3}\right|=0.417$		

*Note*. *P*_*ijk*_: proportion of normal structured organs, where *i* represents individual species, *j* represents individual structures (i.e., muscles), and *k* represent individual parameters of the muscles.

**Table 5:** Analyses of the anatomical data by parametrical *t*-test and nonparametrical SMCA (CAI) [6].

Specimens	Average ratios of length of palmaris longus to tendon length	Primate grouping	CAI (nonparametric analysis)
*Gorilla *	No data in the literature	Apes	0.133
*Pan *	1.78 (±0.04)		0.082
*Pongo *	1.89 (±0.15)		0.036
*Macaca fuscata *	2.37 (±0.12)	Old world primate	0.036
*Callithrix *sp.	2.53 (±0.08)	New world primates	0
*Ateles* sp.	2.53		0
*Sapajus libidinosus *	3.81 (±1.07)		0
*Lemur catta *	4.53 (±0.27)	Prosimii	0
*Propithecus *sp.	5.16 (±0.49)		Reference as more primitive specimen

**Table 6:** Partial agreement between parametric and nonparametric analyses of the palmaris longus of nonhuman primates. The range of values indicates numerical definition of the groups of the primates based on the parametric and nonparametric analyses in Table 5.

Groups of the primates	Definition of groups based on parametric analysis of the ratios (muscle length/tendon length) (range of ratio)	Definition of groups based on nonparametric analysis of the features (range of CAI)
Apes	1.0–2.0	0.100–0.036
Old world primates	2.0–2.5	0.036–0.000
New world primates	2.5–4.0	0.000
Prosimii	4.0–6.0	

**Table 7:** Partial agreement between the two nonparametric analyses (CAI and cladistics) of the palmaris longus in human and nonhuman primates.

	Ce	Hu	Ch	Ba
Nonparametric analysis by CAI				

Pronator quadratus	*Control species *	CAI = 0.375	CAI = 0.000	CAI = 0.000

Flexor digitorum profundus	*Control species *	CAI = 0.000	CAI = 0.063	CAI = 1.000

Flexor pollicis longus	*Control species *	CAI = 0.125	CAI = 0.000	CAI = 0.250

GCAI	*Control species *	0.167	0.020	0.417

Cladistics features of the palmaris longus in features innervation (A), origin (B), insertion (C), and vascularization (D) (0 and 1 indicate absence and presence of primitive features, resp.)				

	(ABCD)	(ABCD)	(ABCD)	(ABCD)

Pronator quadratus	(1111)	(0111)	(1111)	(1111)

Flexor digitorum profundus	(0000)	(0000)	(0000)	(1111)

Flexor pollicis longus	(1101)	(1001)	(1101)	(1111)
